# Cardiac Fibroblast p38 MAPK: A Critical Regulator of Myocardial Remodeling

**DOI:** 10.3390/jcdd6030027

**Published:** 2019-08-07

**Authors:** Neil A. Turner, Nicola M. Blythe

**Affiliations:** Discovery & Translational Science Department, Leeds Institute of Cardiovascular and Metabolic Medicine (LICAMM), School of Medicine, University of Leeds, Leeds LS2 9JT, UK

**Keywords:** cardiac remodeling, extracellular matrix, fibroblast, fibrosis, heart, hypertrophy, inflammation, myofibroblast, p38 MAP kinase, signal transduction, stress-activated protein kinase, transcription factor

## Abstract

The cardiac fibroblast is a remarkably versatile cell type that coordinates inflammatory, fibrotic and hypertrophic responses in the heart through a complex array of intracellular and intercellular signaling mechanisms. One important signaling node that has been identified involves p38 MAPK; a family of kinases activated in response to stress and inflammatory stimuli that modulates multiple aspects of cardiac fibroblast function, including inflammatory responses, myofibroblast differentiation, extracellular matrix turnover and the paracrine induction of cardiomyocyte hypertrophy. This review explores the emerging importance of the p38 MAPK pathway in cardiac fibroblasts, describes the molecular mechanisms by which it regulates the expression of key genes, and highlights its potential as a therapeutic target for reducing adverse myocardial remodeling.

## 1. Introduction

Although once viewed as relatively passive players that solely regulate extracellular matrix (ECM) remodeling, cardiac fibroblasts are now acknowledged as being the primary nodal regulators of multiple aspects of cardiac function under both physiological and pathophysiological conditions [1,2,3,4]. In addition to modulating ECM turnover, cardiac fibroblasts contribute to cardiac inflammation, angiogenesis and cardiomyocyte hypertrophy, and hence are viewed as an important potential therapeutic target for ameliorating adverse cardiac remodeling [5]. Mitogen-activated protein kinase (MAPK) intracellular signaling pathways are central regulators of multiple aspects of cellular function in all tissues, including the heart [6]. The p38 MAPK pathway is activated in response to stress and inflammatory stimuli, and has been shown to play an important role in regulating cardiac remodeling [7,8]. The four kinases that comprise the p38 MAPK family (p38α, β, γ and δ) have differential expression and function in individual cardiac cell types, but the precise role of the p38 pathway in cardiac fibroblasts has not been well explored. The aim of this review is to discuss the importance of p38 MAPK in regulating cardiac fibroblast function in the context of cardiac remodeling, to describe the underlying molecular mechanisms, and to highlight the value of cardiac fibroblast p38 signaling as a potential therapeutic target. 

## 2. Cardiac Fibroblasts and Myocardial Remodeling

Cardiac fibroblasts play a pivotal role in maintaining normal cardiac structure and function, and also contribute to cardiac remodeling in response to myocardial injury or pathologies [1,2,3,4]. The cardiac fibroblast is a remarkably versatile cell type, and is able to adopt alternative phenotypes, proliferate, migrate, secrete a plethora of cytokines and growth factors, and alter the turnover of cardiac ECM [1,2,3,4]. These processes are essential following myocardial infarction (MI) in order to adapt to the altered environment and prevent cardiac rupture. However, if they persist, they can become maladaptive and lead to pathological remodeling, which can advance to heart failure. Classically, cardiac fibroblasts have been understood to differentiate to a myofibroblast phenotype during the remodeling process [9,10], but recent in-depth molecular analyses and lineage-tracing experiments reveal that a continuum of different differentiation states exist in these highly plastic cells following myocardial injury [11,12,13]. 

Cardiac fibroblasts adopt an inflammatory phenotype within the first few days after the MI [14]. Fibroblasts respond to damage-associated molecular patterns (DAMPs) [2]; a collection of intracellular and extracellular molecules whose presence is indicative of a loss of cell membrane integrity, ECM remodeling and a pathological environment. DAMPs are recognized through pattern recognition receptors, including members of the Toll-like receptor (TLR) family, which are coupled to inflammatory signaling pathways resulting in increased secretion of proinflammatory cytokines and chemokines that attract and activate leukocytes in the infarcted area [2].

As the inflammatory signals abate, fibroblasts proliferate and migrate towards the damaged region, and degrade the ECM through upregulation of matrix metalloproteinase (MMP) activity [15], thus facilitating cell migration and angiogenesis. In response to mechanical stimuli and elevated levels of transforming growth factor-β (TGFβ), fibroblasts adopt a highly synthetic and contractile myofibroblast phenotype, characterized by the appearance of cytoplasmic stress fibers containing alpha smooth muscle actin (αSMA) [9,10]. Myofibroblasts originate from resident cardiac fibroblasts, but can also be derived from other cellular sources including endothelial cells, epithelial cells, mesenchymal stem cells, pericytes, smooth muscle cells and bone marrow-derived fibrocytes [9,10]. As myofibroblast numbers increase and they migrate to the site of injury, a pro-fibrotic environment is created. Increased ECM deposition by myofibroblasts is the net result of increased type I and III collagen synthesis, decreased MMP activity and an increased activity of the tissue inhibitors of metalloproteinases (TIMPs), the native inhibitors of MMPs [16]. Myofibroblasts undergo apoptosis if ECM tension is restored, but if a mechanical load persists, such as with chronic hypertension, there is prolonged myofibroblast activity, and fibrosis occurs [17]. Fibrosis results in stiffening of the heart, impairment of electrical conductivity and reduced pumping capacity and oxygen diffusion, thus exacerbating adverse remodeling, and leading to heart failure progression. In addition to driving fibrosis, cardiac fibroblasts are now recognized as being important regulators of cardiac hypertrophy [18,19]. Fibroblasts synthesize a range of paracrine signaling molecules, including growth factors, cytokines and microRNAs, that can act to stimulate hypertrophy of cardiomyocytes [18,19].

The multifactorial functions of cardiac fibroblasts in myocardial remodeling make them an attractive target therapeutically. However, inadequate understanding of the regulatory mechanisms governing this cell type so far hinders the development of therapies targeting the cardiac fibroblast and its pathological involvement in disease progression. 

## 3. p38 MAP Kinase

### 3.1. MAP Kinase Signaling Cascades

**Figure 1:**
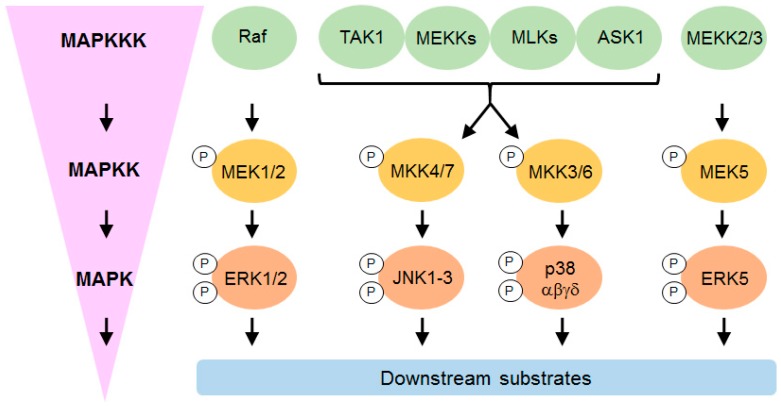
Canonical MAPK signaling. Schematic simplified representation of the different classical MAP kinase pathways in mammals. The mitogen-activated protein kinase (MAPK) pathways consist of a cascade of three kinases which phosphorylate (indicated by P) and activate each other sequentially. Mitogens, cytokines, and cellular stresses act as the stimulus to promote the activation of these MAPK pathways. MAPKs regulate cellular function by phosphorylating downstream substrates in the nucleus (e.g., transcription factors), cytosol, cytoskeleton, mitochondria and plasma membrane. MAPK: Mitogen-activated protein kinase; MAPKK: Mitogen-activated protein kinase kinase; MAPKKK: Mitogen-activated protein kinase kinase kinase.

The MAPKs are a large family of kinases associated with vital cell functions such as gene regulation, proliferation, differentiation, mobility, survival and death [20]. There are four classical MAPK signaling cascades: Extracellular signal-regulated kinases (ERK1/2), c-Jun N-terminal kinases (JNK1, 2 and 3), p38 mitogen-activated kinases (p38α, β, γ and δ) and ERK5 (Figure 1). Further atypical MAPKs have also been identified (ERK3/4, ERK7 and Nemo-like kinase). 

The classical MAPK signaling cascade involves a number of sequential phosphorylation events which commence with a MAP kinase kinase kinase (MAPKKK) phosphorylating a MAP kinase kinase (MAPKK) on specific serine/threonine residues, which in turn leads to dual phosphorylation of the MAPK on specific threonine and tyrosine residues (Figure 1). This dual phosphorylation always occurs in a Thr-X-Tyr motif, where X is either a Glu (ERK), Pro (JNK) or Gly (p38) residue in the regulatory loop [20]. Activated MAPKs continue the cascade in order to alter different aspects of cell function; they can exert their effects directly by phosphorylating downstream substrates such as transcription factors, or indirectly by activating downstream kinases, which in turn phosphorylate their own substrates.

Despite the apparently simple architecture of this pathway, MAPKs are capable of responding to a plethora of different stimuli. The JNK and p38 MAPKs are activated by environmental stresses and inflammatory stimuli (e.g., ultraviolet light, oxidant stress, osmotic shock, infection and cytokines [21]), and are collectively known as stress-activated protein kinases (SAPKs). The SAPKs mediate cell death, differentiation and inflammatory responses. In contrast, the ERK1/2 pathway plays important roles in proliferation, differentiation and survival, and is activated only to a smaller degree by stress stimuli; rather, growth factors, serum, and phorbol esters are those which strongly activate the pathway [6]. ERK5 is the final MAPK subfamily and the least studied of the four; it is activated by both growth and stress stimuli [22]. This review will focus exclusively on p38 MAPK, a kinase that has long been recognized as a driver of myocardial remodeling, and whose role in modulating cardiac fibroblast function is becoming more appreciated. The ERK1/2, JNK and ERK5 families will not be discussed further, but the reader is referred to some thorough reviews on the roles of these kinases in the heart [6,23].

### 3.2. p38 MAP Kinase Signaling

#### 3.2.1. p38 Subtypes and Their Activation

Four different p38 MAPK isoforms have been identified; p38α (MAPK14/SAPK2a), p38β (MAPK11/SAPK2b) and the more distantly related p38γ (MAPK12/SAPK3) and p38δ (MAPK13/SAPK4). The four isoforms share structural homology (particularly between p38α and β, and between p38γ and δ) and substrate similarities. P38α is the most well-characterized isoform and is ubiquitously expressed, whereas the other subtypes display differential tissue and cell-type expression. Hence, most studies that refer to p38 MAPK most likely relate to the α subtype. P38α and β can be inhibited by pyridinyl imidazole drugs (e.g., SB203580, SB202190), whereas the diaryl urea compound BIRB-0796 inhibits all four p38 subtypes [24]. A number of orally-active p38α/β inhibitors have been developed for clinical use, including losmapimod (GW85655) and RWJ-67657 [24]. 

Various upstream kinases are involved in the phosphorylation cascades leading to p38 activation, including MST1, MEKK1–4, MLK3, TAK1 and ASK1 at the MAPKKK level, and MKK3/4/6 at the MAPKK level (Figure 1). P38 can also be activated by at least two non-canonical mechanisms. One of these is through TAB1 (TGF-β-activated protein 1 (TAK1)-binding protein 1), which binds to p38α MAPK and leads to its autophosphorylation [25,26]. This mechanism contributes to injury during myocardial ischemia [25]. The other method has only been observed in T-cells stimulated through the T-cell antigen receptor (TCR), and involves the tyrosine kinase ZAP70 [27].

#### 3.2.2. Modulation of Gene and Protein Expression

The p38 pathway is able to modulate gene and protein expression through multiple complementary methods, including the regulation of chromatin structure, gene transcription, mRNA stability and protein translation. Firstly, p38 can modulate chromatin structure by influencing DNA methylation [28] and phosphorylating histones e.g., via the activation of mitogen- and stress-activated protein kinase 1 (MSK1) [29]. Secondly, p38 can directly alter gene transcription by phosphorylating key target residues on specific transcription factors to modulate their activity. These include ATF1/2/6, C/EBPα, CHOP, CREB, c-MYC, c-FOS, ELK1, GATA4, MEF2A/C/D, NFAT, SRF and STAT1 [8,24]. P38 can also induce an expression of the early response gene c-FOS, a component of the AP-1 transcription factor, via ELK1 activation [30]. Thirdly, the p38 pathway is a strong regulator of the stability and decay of specific mRNAs through the phosphorylation of specific RNA binding proteins, including the mRNA stabilizing factor HuR (human antigen R) and the mRNA destabilizing factor TTP (tristetraprolin) [31,32]. HuR and TTP compete with one another to regulate mRNA stability through binding to AU-rich elements (AREs) in the 3′ untranslated regions (3′UTRs) of specific mRNAs. Phosphorylation of HuR increases the stability of specific mRNAs, whereas phosphorylation of TTP reduces mRNA decay. Thus, p38-mediated phosphorylation of either or both of these RNA binding proteins results in an increase in steady state mRNA levels. Although the precise mechanism by which p38 phosphorylates the regulators of mRNA stability remains unknown, most of the evidence suggests that MAPK-activated protein kinase 2 (MK2), a p38 substrate, is the kinase responsible [31,33]. Finally, the p38 pathway can regulate protein translation through the phosphorylation of MAPK-interacting serine/threonine-protein kinase 1 (MNK1) that in turn regulates the eukaryotic initiation factor 4E (eIF4E) [34].

#### 3.2.3. MAPK-Activated Protein Kinases

MK2 and MK3 are stimulated by the p38 module in response to stress stimuli [35]. Targeted deletion of the mouse *Mk2* gene provides evidence that, although p38 regulates a diverse set of substrates, MK2 is fundamental in p38-dependent biological processes [36]. Both MK2 and MK3 can be activated by p38α, but the expression level and activity of MK2 is considerably higher than that of MK3 [35]. The substrate range of both enzymes includes proteins involved in cytokine production, endocytosis, reorganization of the cytoskeleton, cell migration, cell cycle control, chromatin remodeling and transcriptional regulation [35]. 

MK5 is more distantly related to MK2/3, but it has been shown that, under conditions of overexpression, p38 phosphorylates MK5 in response to cellular stress [37]. Despite this, some evidence suggests that MK5 is not a physiological p38 substrate, as it is not activated by classical p38 stimuli, and no interaction between endogenous MK5 and p38 has been reported [38]. MK5 has more recently been shown to be a physiological substrate of ERK3/4 [39].

#### 3.2.4. MAPK Phosphatases

The balance between kinase activation and inactivation adds another layer of regulation by which the MAPK signaling is tightly controlled. Protein phosphatases remove phosphate groups added by kinases, thus determining the magnitude, duration and localization of kinase activation. Dual-specificity phosphatases (DUSPs), formerly termed MAPK phosphatases (MKPs), can either inactivate a single class of MAPK (e.g., DUSP6/MKP3 specifically inactivates ERK), or they can regulate more than one MAPK pathway (e.g., DUSP1/MKP1 dephosphorylates ERK, JNK and p38) [6,40,41]. The phosphatases that can inactivate p38 MAPK include the nuclear DUSPs 1, 2 and 4, the nuclear and cytosolic DUSPs 8, 10 and 16, and protein Ser/Thr phosphatases such as PP2C [6,40,41]. 

## 4. p38 MAP Kinase and Regulation of Cardiac Remodeling

### 4.1. In Vitro and In Vivo Studies

There is a wide body of in vitro and pre-clinical in vivo evidence that points to a detrimental role of p38 MAPK in cardiac remodeling, based largely upon the pharmacological p38 inhibition and transgenic/viral manipulation of p38 subtypes or upstream activators such as MKK3 and MKK6. In the interests of space, a full review of this literature is not possible here, but the reader is referred to a number of excellent reviews on this topic that give a detailed evaluation of the underlying primary literature [7,8,42,43]. Inhibiting the p38 pathway may therefore be a viable therapeutic strategy to ameliorate the adverse myocardial remodeling associated with MI, hypertrophy and heart failure [7]. Interestingly, many of the effects of p38 appear to be manifested through the downstream kinase MK2, and in addition, the administration of MMI-0100, a cell-permeable peptide inhibitor of MK2, has been shown to mimic the effects of p38 inhibition and improve cardiac remodeling [44,45,46]. 

The p38 pathway contributes to cardiac and vascular inflammation through the induction of inflammatory cytokines and reactive oxygen species [43,47]. In addition to effects on immune cells, p38 has direct effects on cardiac cell types. Ectopic overexpression studies suggest that p38β stimulates cardiomyocyte hypertrophy, whereas p38α stimulates myocyte apoptosis [48,49]. However, in vivo studies inhibiting or knocking out these subtypes selectively in cardiomyocytes fail to improve cardiac function in hypertrophy models, and in some cases exacerbate the problem [50,51,52,53]; in stark contrast to pre-clinical studies, where pharmacological p38 inhibitors are effective [7,8,42,43]. An obvious difference between these two types of study is that the kinase inhibitors affect all cardiac cell types (myocytes, fibroblasts, endothelial cells, inflammatory cells), whereas the genetic targeting approaches target just cardiomyocytes. Hence, it is important to appreciate the cell-specific roles of these p38 subtypes in regulating cardiac remodeling. 

Although the vast majority of our knowledge on p38 in the heart relates to p38α, and to a lesser extent p38β, the roles of p38γ and p38δ remain elusive. However, recent evidence suggests that the γ and δ subtypes are important in both developmental and pathological hypertrophy via the modulation of the mTOR pathway [54]. As discussed above, the spatiotemporal nature of p38 signaling is regulated by phosphatases, particularly members of the DUSP family. DUSPs have been shown to play important regulatory roles in cardiac hypertrophy and remodeling [41], and DUSP1/4 double knockout leads to unrestrained p38 activation and induction of cardiomyopathy [55]. 

### 4.2. Clinical Studies

The development of p38 MAPK inhibitors for use in the clinic was originally limited to patients with chronic inflammatory diseases but, more recently, there has been interest in p38 inhibition in cardiovascular diseases [7,24,43]. 

The orally active p38α/β inhibitor losmapimod was evaluated for its effect on inflammation and infarct size in patients with non-ST-segment elevation MI in the SOLSTICE study [56]. This randomized phase 2 multicenter trial demonstrated the beneficial effects of losmapimod, including improved LV function and a non-significant trend towards reduced infarct size in losmapimod-treated subjects. However, the follow up LATITUDE-TIMI 60 phase 3 multicenter trial failed to show a reduction in a risk of major ischemic cardiovascular events after 12 weeks, despite a reduction in inflammation and levels of wall stress [57]. Improved knowledge of the role of p38 in the cardiovascular system could help fine tune therapeutic p38 inhibition for the treatment of cardiovascular disease in the future. 

## 5. p38 MAP Kinase and Regulation of Cardiac Fibroblast Function

### 5.1. In Vitro Studies

Human cardiac fibroblasts express relatively high levels of p38α, with lower levels of p38γ and p38δ, and very low or undetectable levels of p38β [58]. A similar profile of subtype expression is evident in whole heart tissue [59,60]. The importance of p38 in regulating cardiac fibroblast function is derived largely from in vitro cell culture studies using human, rat and mouse cells. Stimuli that induce p38 signaling in cardiac fibroblasts include inflammatory cytokines (e.g., IL-1, TNFα), profibrotic cytokines (e.g., TGF-β), DAMPs, G-protein coupled receptor agonists (e.g., angiotensin II [Ang II], β-adrenoceptors), ischemia and mechanical stretch [2,3,58,61].

Most of our knowledge on p38 signaling in cardiac fibroblasts relates to the α subtype, as this is the most highly expressed subtype, and (along with p38β) is the target of the pyridinyl imidazole class of inhibitors including SB203580 and RWJ-67657. In contrast, the functional importance of the other subtypes in cardiac fibroblasts is largely unexplored. Pharmacological and molecular interference studies ascribe a key role for fibroblast p38α in regulating the mRNA expression and protein secretion of a range of proinflammatory cytokines (e.g., IL-1, IL-6, IL-8, TNFα) [58], enabling cardiac fibroblasts to contribute to local inflammation after cardiac injury [2,14]. The p38 pathway also modulates ECM turnover through controlling the expression of several MMPs expressed by cardiac fibroblasts (e.g., MMPs 1, 3, 9) [15,58], as well as type I collagen [62,63,64] and TIMP-1 [65]. Additionally, the p38 pathway regulates the proliferation, migration and differentiation of cardiac fibroblasts. These elements will be explored in more detail below.

#### 5.1.1. Proinflammatory Cytokines

Induction of inflammatory gene transcription is the initial specific step that leads to increased mRNA levels of particular cytokines, but the duration of this response is under tight control by post-translational mechanisms, including the regulation of mRNA stability [32]. The promoter regions of genes encoding inflammatory cytokines have binding sites for a limited set of transcription factors, including NFκB, AP-1, C/EBPβ and Ets family members (e.g., ELK1); all of which can be activated downstream of the p38 pathway. Many inflammatory cytokines have characteristically short half-lives, enabling the rapid dampening of inflammatory responses in the absence of prolonged stimulation. For example, the half-life of *TNF* mRNA is only 30 min in human cardiac fibroblasts [66]. A number of proinflammatory cytokines that are secreted by cardiac fibroblasts (e.g., IL-1, IL-6, IL-8, TNFα) are regulated at the post-transcriptional level by p38-induced MK2-mediated modulation of RNA-binding proteins, including TTP and HuR [33]. Studies on cultured human cardiac fibroblasts identify roles for p38 in inducing the mRNA expression of TNFα, IL-1β and IL-6, whereas p38 appears to regulate IL-8 at the protein synthesis/secretion level [66,67,68]. IL-6 is discussed further in the next section.

#### 5.1.2. Hypertrophic Factors

A paradigm that has emerged over the last few years is that cardiac fibroblasts are important modulators of cardiac hypertrophy through their secretion of specific paracrine hypertrophic factors, including growth factors, cytokines and microRNAs [18,19]. Amongst the list of paracrine factors identified to date, several can be modulated by the p38 pathway, including fibroblast growth factor (FGF)-2 [69,70], insulin-like growth factor (IGF)-1 [71], TGF-β [72] and IL-6 (see below).

Some of the most compelling evidence for a p38-regulated paracrine mediator of cardiomyocyte hypertrophy produced by cardiac fibroblasts relates to IL-6. IL-6 is a pleiotropic cytokine that has proinflammatory, anti-inflammatory and fibrotic roles in the heart, depending on the context and duration of its effect [73]. In vitro studies have shown that IL-6 is secreted from cardiac fibroblasts in response to several well-known hypertrophic stimuli, including β-adrenergic receptor stimulation and Ang II [1]. IL-6 can directly induce cardiomyocyte hypertrophy in vitro [74,75] and Ang II- or phenylephrine-induced cardiomyocyte hypertrophy is impaired in myocytes isolated from IL-6 knockout mice [74], suggesting that IL-6 secretion is necessary for the induction of myocyte hypertrophy by these stimuli. 

**Figure 2:**
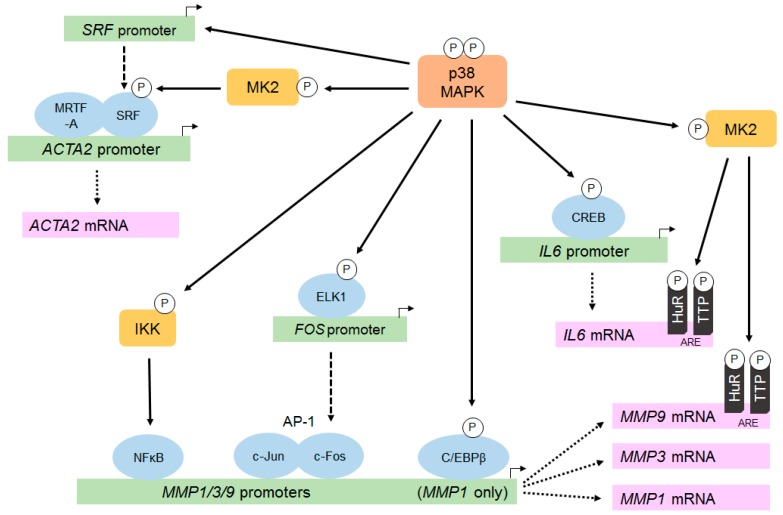
Molecular mechanisms by which p38 regulates the expression of MMPs, αSMA and IL-6 in cardiac fibroblasts. The p38 pathway is important for upregulating the expression of specific proteins in cardiac fibroblasts, including matrix metalloproteinases (MMPs), α-smooth muscle actin (αSMA/ACTA2) and interleukin-6 (IL-6). Modulation occurs at multiple levels, including the regulation of gene transcription and mRNA stability. P38 activation leads to phosphorylation and activation of specific transcription factors (via both direct and indirect mechanisms), and can also increase stability of specific mRNAs through the phosphorylation of mRNA stabilizing factors (e.g., HuR) and mRNA destabilizing factors (e.g., TTP). See main text for more details.

Evidence from numerous in vitro cardiac fibroblast studies points to a pivotal role for p38α in stimulating *IL6* mRNA expression and protein secretion in response to inflammatory cytokines and neurohumoral stimuli (reviewed in [24]). The p38 pathway is an important inducer of IL-6 expression in the heart, acting via both transcriptional and post-transcriptional (e.g., increased mRNA stability) mechanisms [47,76] (see Figure 2 for a summary). For example, Ang II-induced, p38-dependent *Il6* transcription in rat cardiac fibroblasts involves the phosphorylation of the CREB transcription factor and binding to specific CRE sites in the *IL6* gene promoter [77]. In addition to transcriptional effects, p38α can stabilize *IL6* mRNA via multiple AREs in the 3′UTR of the *IL6* transcript [78], and a role for TTP phosphorylation in this process has been established in cancer cells [79]. Localized adenovirus-mediated gene transfer of activated MKK3/p38α in rat hearts revealed IL-6 to be a central signaling molecule which was implicated in the regulation of numerous p38-sensitive genes through the modulation of several transcription factors including GATA-4, AP-1, SRF and NFκB [76]. 

Recent fibroblast-targeted transgenic mouse studies provide support for fibroblast-derived IL-6-driving cardiac hypertrophy in vivo [80], with p38α playing a key role [81]. Further studies in murine cardiac hypertrophy models using animals with cardiac fibroblast-specific deletion of the *IL6* gene would be useful to confirm this hypothesis.

#### 5.1.3. Matrix Metalloproteinases

MMP expression and activity can be regulated at multiple levels including transcription, mRNA stability, translation, secretion, zymogen activation and endogenous inhibition by TIMPs [82]. The p38 pathway impacts on MMP activity predominantly by increasing transcription and mRNA stability (see below), although it can also influence TIMP expression [65]. Several MMPs expressed by cardiac fibroblasts are regulated by p38, including MMP-1, MMP-3 and MMP-9 [15,58]. The potential underlying molecular mechanisms are summarized in Figure 2. The promoter regions of the genes encoding MMPs 1, 3 and 9 contain common binding sites for AP-1 and NFκB [15,83]; transcription factors that can be activated in response to p38 activation via increased FOS expression and IKK phosphorylation, respectively. In addition to these sites, the *MMP1* promoter contains a binding site for the CCAAT/enhancer binding protein-β (C/EBP-β), a transcription factor that is directly phosphorylated and activated by p38 [84]. 

IL-17-induced *MMP1* mRNA expression in human cardiac fibroblasts has been shown to occur in a p38- and ERK-dependent manner involving transcription factor binding at the AP-1, NFκB and C/EBP-β sites [85]. In addition to inducing *MMP1* gene transcription, p38 can also increase *MMP1* mRNA levels by increasing *MMP1* mRNA stability [86], although the mechanism is unclear.

Cytokine-induced MMP-3 expression is mediated via p38α in human cardiac fibroblasts [58,87] and dermal fibroblasts [86], but appears to be p38-independent in rat cardiac fibroblasts [88]. In human dermal fibroblasts, cytokine-induced increases in *MMP3* mRNA levels were due to the stabilization of *MMP3* mRNA, rather than increased transcription [86]. However, as with MMP1 above, the underlying regulatory mechanisms are unclear, as *MMP3* mRNA does not contain consensus AREs in its 3′UTR.

Activation of p38 increases *MMP9* gene transcription through NFκB and AP-1 transcription factors binding to the gene promoter [89,90]. IL-1-induced *MMP9* mRNA expression is p38-dependent in rat cardiac fibroblasts [88], although it appears to be p38-independent in human cardiac fibroblasts [58,87]. In addition to modulating gene transcription, p38-mediated activation of the downstream kinase MK2 has been shown to increase *MMP9* mRNA stability [91]. The 3′UTR of *MMP9* mRNA contains several AREs [92] and p38 activation is able to increase *MMP9* mRNA stability through the phosphorylation of TTP [79] and by targeting HuR [93]. 

#### 5.1.4. α-Smooth Muscle Actin

Transcription of the *ACTA2* gene, encoding α-SMA, requires the formation of nuclear complexes comprising the serum-response factor (SRF) and myocardin-related transcription factor-A (MRTF-A) [94]. The p38 pathway induces *ACTA2* expression through at least two complementary mechanisms (summarized in Figure 2). Firstly, p38 is critical for TGF-β-induced SRF gene expression; a prerequisite for the formation of SRF/MRTF-A complexes and *ACTA2* gene transcription [95]. Secondly, the p38 downstream kinase MK2 phosphorylates and activates SRF, thereby further contributing to increased *ACTA2* transcription [96]. 

In rat cardiac fibroblasts, the pyridinyl imidazole p38 inhibitors RWJ-67657 and SB203580 have been shown to abrogate αSMA expression [62,97], confirming a key role for p38α in regulating αSMA expression in these cells. Furthermore, αSMA expression was reduced in mouse embryonic fibroblasts (MEFs) with a conditional deletion of p38α, and this was overcome by the ectopic expression of SRF, confirming that p38α lies upstream of SRF and αSMA expression in fibroblasts [98].

#### 5.1.5. Proliferation, Migration and Myofibroblast Differentiation

P38α is also important for cardiac fibroblast proliferation, migration and myofibroblast differentiation [24]. Specific evidence for a role of p38 in myofibroblast differentiation and fibrosis comes from studies showing that TGF-β-induced expressions of collagen I and αSMA are attenuated by p38 inhibition in rat and mouse cardiac fibroblasts [62,63,64]. MEFs with conditional p38α deletion impair the capability to contract collagen gels and exhibit reduced strain-induced αSMA expression [98]. The ability of p38 to promote cardiac myofibroblast differentiation and migration appears to be mediated via its downstream substrate, MK2 [44], and similar conclusions have been drawn in studies using fibroblasts from other sources [99,100]. A role for the inactivation of the phosphatase DUSP1/MKP1 (and hence enhanced p38 and ERK activation) has been proposed as a mechanism by which IL-17A stimulates cardiac fibroblast proliferation [101]. 

### 5.2. In Vivo Studies

Methods for genetically modifying cardiac fibroblasts in vivo have lagged well behind those for targeting cardiomyocytes, and establishment of such models has been an important goal for progressing the cardiac fibroblast field for many years [24]. A number of fibroblast-targeted Cre-lox models have now been well characterized in lineage-tracing experiments and shown to target fibroblasts and/or myofibroblasts in the heart, as well as in other tissues. The models that have been most widely utilized are driven by promoter/enhancer regions from the *Col1a2* [102], *Postn* [103,104] and *Tcf21* [103,104,105] genes. When combined with tamoxifen-inducible forms of Cre, these have been employed to manipulate gene expression (knockout and overexpression) conditionally and specifically in the fibroblast (*Col1a2*, *Tcf-21*) or myofibroblast (*Postn*) populations of the murine heart [81,98,106,107,108,109]. 

#### 5.2.1. Fibroblast-Specific Targeting of p38α

Several recent studies exploit these in vivo fibroblast-targeting strategies to define a critical role for cardiac (myo)fibroblast p38α in modulating hypertrophic and fibrotic cardiac remodeling. Molkentin and colleagues developed both fibroblast- and myofibroblast-selective *Mapk14* knockout mouse models to investigate the contribution of p38α to cardiac fibrosis after myocardial injury [98]. Mice with a conditional deletion of p38α in resident fibroblasts (*Tcf21*-Cre) were more prone to cardiac rupture with 100% mortality observed after experimental MI (permanent LAD ligation) and 50% mortality observed after ischemia-reperfusion injury (transient LAD ligation), suggesting cardiac fibroblast p38α is important for effective scar formation. Fibroblast-specific p38α-null animals that survive the ischemia-reperfusion procedure exhibited less fibrosis and improved diastolic function. When p38α knockout was induced exclusively in myofibroblasts (*Postn*-Cre), there was a reduction in the MI scar and fibrotic area and a reduction in myofibroblast numbers, without impacting cardiac function [98]. Cardiac fibrosis and hypertrophy induced by chronic neurohumoral stimulation were also reduced in mice with a myofibroblast-specific deletion of p38α. Conversely, fibroblast-specific activation of p38 (by conditional *Tcf21*-directed expression of constitutively active MKK3) results in increased fibrosis in the heart (and other tissues), expansion of the fibroblast number and increased myofibroblast differentiation; effects that were exacerbated following MI or chronic neurohumoral stimulation [98]. 

In addition to this role in regulating fibrosis and myofibroblast differentiation, we recently reported that fibroblast-specific p38α is also important for stimulating cardiac hypertrophy in a mouse model of chronic β-adrenergic stimulation [81]. Fibroblast-specific deletion of p38α (using a tamoxifen-inducible *Col1a2*-Cre model) significantly improves cardiac function after chronic β-adrenergic stimulation and reduces the resultant increase in cardiac mass and cardiomyocyte size compared to littermate controls. In vitro mechanistic studies indicated an important role for IL-6 as a p38α-induced paracrine factor to be released by fibroblasts in response to tissue damage that could subsequently stimulate cardiomyocyte hypertrophy [81]. A reduction in cardiac hypertrophy was also noted in the fibroblast-specific p38α knockout hearts in the study by Molkentin and colleagues [98], and we also observed a reduction in cardiac fibrosis in our study [81]. Thus, these two complementary independent studies using different fibroblast-specific targeting strategies offer strong evidence that p38α is a critical regulator of cardiac fibrosis and hypertrophy following MI or chronic neurohumoral stimulation. It would be interesting to evaluate the impact of fibroblast-specific p38α knockout in additional pathological models, including chronic pressure- and volume-overload.

#### 5.2.2. Fibroblast-Specific Targeting of Other Components of the p38 Pathway

Further supporting evidence of the importance of the fibroblast p38 pathway in cardiac remodeling comes from a non-biased transcriptomic approach that identifies the ATF3 transcription factor as being cardioprotective in hypertensive cardiac remodeling [109]. Cardiac fibroblasts were shown to be the primary cardiac cell type that express ATF3, and fibroblast-specific ATF3 deletion (*Col1a2*-Cre) attenuated hypertensive cardiac fibrosis and hypertrophy induced by Ang II. Further detailed bioinformatic and integrated transcriptomics revealed that ATF3 exerts its cardioprotective effects by repressing the *Map2k3* locus (the gene encoding MKK3), which in turn reduces the p38 activity in cardiac fibroblasts [109]. Conversely, fibroblast-specific overexpression of ATF3 (driven by *Col1a2*-Cre) improves cardiac function and remodeling after MI [110], thus confirming that activation of cardiac fibroblast ATF3 represents a potential therapeutic strategy for improving cardiac remodeling and function. 

MK5 was originally identified as a downstream substrate of p38α/β, and subsequently the atypical kinases ERK3 and ERK4 [37,39]. Despite similar expression levels of *MK5* mRNA in cardiac myocytes and fibroblasts [111], the protein expression of MK5 appears to be restricted to fibroblasts [112], suggesting cell-specific MK5 translational regulation. Global knockdown of MK5, which therefore effectively targets the fibroblast population in the heart, attenuates both hypertrophy and cardiac dysfunction in response to chronic pressure overload [112]. Thus, the fibroblast-selective inhibition of MK5 appears to be cardioprotective, in agreement with the above p38-focused studies. However, it remains unclear whether p38 is a genuine physiological activator of MK5 [38], and in mouse heart MK5 forms a stable signaling complex only with ERK3 (not p38α or ERK4) [113]. Hence caution is warranted when interpreting MK5 data in terms of the p38 pathway.

Conditional deletion of MK2 has not been studied in cardiac fibroblasts in vivo, but the *Col1a2*-Cre model has been used to delete MK2 in a study focused on lung fibrosis [99]. Fibroblast-specific MK2 deletion attenuated bleomycin-induced lung fibrosis in mice, and similar results were obtained following the administration of a cell-permeable peptide inhibitor of MK2, MMI-0100 [99]. 

#### 5.2.3. MicroRNAs

**Figure 3:**
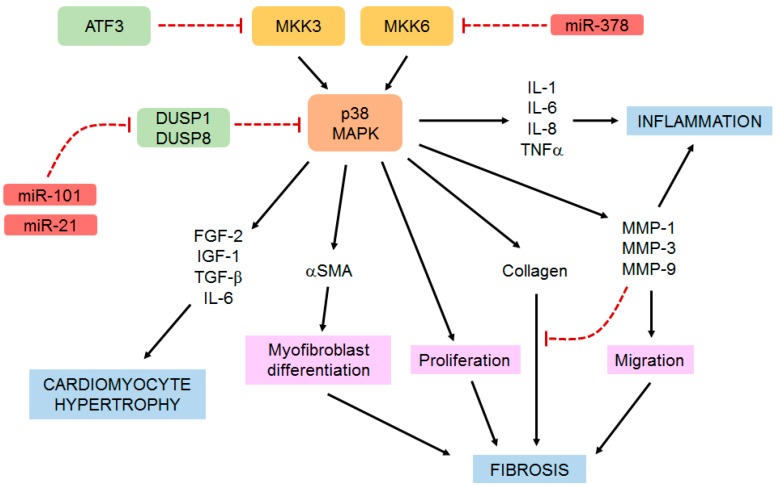
Central role of p38 in regulating cardiac fibroblast function. Cardiac fibroblast p38 is important for regulating several key aspects of cardiac remodeling, including inflammation, fibrosis and cardiomyocyte hypertrophy through a variety of molecular and cellular mechanisms. See the main text for more details.

MicroRNAs are small non-coding RNAs that regulate the expression of specific proteins by promoting mRNA degradation or inhibiting protein translation [114]. Several miRNAs are known to play important roles in regulating cardiac fibroblast phenotype and function [10,115], and some mediate their effects through their modulation of the p38 MAPK pathway (Figure 3). For example, miR-101 has been shown to be important for IL-17A-induced cardiac fibroblast proliferation and migration via a mechanism involving the inhibition of DUSP1/MKP1 expression, thus increasing p38 and ERK activity [101]. Similarly, miR-21 promotes high glucose-induced cardiac fibroblast proliferation and collagen synthesis by suppressing DUSP8 expression and activating JNK and p38 MAPK [116]. In another example, miR-378 released from cardiomyocytes was shown to induce anti-fibrotic effects by targeting MKK6 in cardiac fibroblasts and reducing p38 MAPK phosphorylation [117].

Recently we identified a small number of miRNAs (miRs 21a, 30d, 208b, 214 and 224) that were up- or down-regulated with cardiac remodeling in control mice following chronic β-adrenergic stimulation, but not in fibroblast-specific p38α knockout mice [81]. Further studies will be needed to evaluate the roles of these p38-regulated miRNAs in cardiac fibroblasts. 

## 6. Conclusions and Future Perspectives

Taken together, the above in vitro and in vivo studies provide persuasive evidence that the p38 pathway (particularly p38α) in cardiac fibroblasts is a critical regulator of myocardial remodeling, and hence a viable therapeutic target. The central role of p38 in regulating cardiac fibroblasts functions that are pertinent to cardiac remodeling is summarized in Figure 3. 

The advent of in vivo fibroblast-specific targeting approaches has enabled the identification of cardiac fibroblast-specific p38α as being a critical mediator of hypertrophic and fibrotic remodeling in a variety of pathological scenarios [81,98]. However, translating these pre-clinical genomic studies into effective therapeutic treatments for cardiovascular patients is challenging. Given the disappointing results of the LATITUDE-TIMI 60 study in which global p38 inhibition with losmapimod fails to improve outcomes six months after MI [57], alternative approaches that target upstream or downstream components of the p38 pathway, or act in a cell-selective manner, may be warranted. Whilst cell-selective targeting of p38α is unrealistic given the ubiquitous nature of its expression, several components of the p38 signaling pathway are selectively expressed in cardiac fibroblasts, including ATF3 [109] and MK5 [112]. 

This may allow pharmacological agents to be developed that target upstream (e.g., ATF3 activation) or downstream (e.g., MK5 inhibition) molecules in the p38 signaling pathway specifically within the cardiac fibroblast.

Another potential strategy for cell-specific therapies is to target microRNAs [118]. As many microRNAs show differential cellular expression and regulation, identifying and targeting cardiac fibroblast-specific microRNAs that regulate the p38 pathway could enable more targeted therapies. 

In conclusion, the p38 MAPK signaling node is gaining recognition as a critical regulator of a variety of cardiac fibroblast functions that contribute to adverse myocardial remodeling, including hypertrophy and fibrosis. Future therapeutic strategies that specifically target cardiac fibroblast p38 signaling may offer benefits in reducing the consequences of detrimental cardiac remodeling.
